# Ectonucleotidase CD39 is highly expressed on ATLL cells and is responsible for their immunosuppressive function

**DOI:** 10.1038/s41375-020-0788-y

**Published:** 2020-03-20

**Authors:** Yasuhiro Nagate, Sachiko Ezoe, Jiro Fujita, Daisuke Okuzaki, Daisuke Motooka, Tomohiko Ishibashi, Michiko Ichii, Akira Tanimura, Masako Kurashige, Eiichi Morii, Takuya Fukushima, Youko Suehiro, Takafumi Yokota, Hirohiko Shibayama, Kenji Oritani, Yuzuru Kanakura

**Affiliations:** 1 Department of Hematology and Oncology, Osaka University Graduate School of Medicine, Suita, Japan; 2 Department of Environmental Space Infection Control, Osaka University Graduate School of Medicine, Suita, Japan; 3 Genome Information Research Center, Research Institute for Microbial Diseases, Osaka University, Suita, Japan; 4 Department of Vascular Physiology, National Cerebral and Cardiovascular Center Research Institute, Suita, Japan; 5 Department of Pathology, Osaka University Graduate School of Medicine, Osaka University, Suita, Japan; 6 Laboratory of Hematoimmunology, School of Health Sciences, Faculty of Medicine, University of the Ryukyus, Nishihara, Japan; 7 Department of Hematology, National Kyushu Cancer, Fukuoka, Japan; 8 Department of Hematology, Graduate School of Medical Sciences, International University of Health and Welfare Hospital, Narita, Japan

**Keywords:** Acute lymphocytic leukaemia, Immunoediting

## Abstract

Adult T-cell leukemia/lymphoma (ATLL) patients have an extremely poor prognosis, partly due to their immunosuppressive state. The majority of ATLL patients have leukemic cells with phenotype similar to Tregs, prompting suggestions that ATLL cells themselves have immunosuppressive functions. In this study, we detected CD39 expression on ATLL cells, particularly frequent on aggressive subtypes. CD39 and CD73 convert extracellular adenosine triphosphate (ATP) into adenosine, a key player in Tregs’ immunosuppression. In vitro culture, both CD39^+^ ATLL cells and normal Tregs converted rapidly extracellular ATP to AMP, which was disturbed by CD39 inhibitors, and was negated in the CD39 knockout MJ cell line. The proliferation of cocultured CD4^+^/CD8^+^ normal T cells was suppressed by CD39^+^ MJ cells, but not by CD39 knockout MJ cells. Supplemented ATP was exhausted by an EG7-OVA T-cell line with stable CD39 induction, but not by mock. When these cell lines were subcutaneously transplanted into murine flanks, Poly(I:C) peritoneal administration reduced tumor size to 1/3 in mock-transplanted tumors, but not in CD39 induced tumors. Overall, we found that ATLL cells express CD39 at a high rate, and our results suggest that this helps ATLL cells escape antitumor immunity through the extracellular ATPDase-Adenosine cascade. These findings will guide future clinical strategies for ATLL treatment.

## Introduction

Adult T-cell leukemia/lymphoma (ATLL) is a mature T-cell neoplasm that has been linked to the human T-cell lymphotropic virus HTLV-1 [1, 2]. Despite intensive research, the prognosis of ATLL remains poor. The acute and lymphoma subtypes are extremely aggressive, and most patients die within 1 year after diagnosis [3, 4], mainly due to chemotherapy resistance and severe immunosuppression [5]. Patients with ATLL are often susceptible to opportunistic infections, such as cytomegalovirus, human herpes virus-6, and *Pneumocystis jirovecii* [6].

The etiology of immunosuppression in ATLL patients is unclear. HTLV-1 primarily infects CD4^+^ T cells and other blood cells, and most ATLL cells have the phenotype CD4^+^CD25^+^CCR4^+^FoxP3^+^. This is the same phenotype of the immunosuppressive T-cell subset termed regulatory T cells (Tregs) [7, 8], prompting suggestions that ATLL cells originate from Tregs. However, it remains controversial whether ATLL cells themselves have immunosuppressive functions, with some studies reporting that ATLL cells have suppressive functions [9, 10], and others finding that they do not [11, 12].

Recent reports describe the phenotypic and functional categorization of human FoxP3^+^CD4^+^ cells into three groups, among which the CD45RA^−^FoxP3^low^ subset comprises non-Tregs without immunosuppressive function [13]. It was proposed that immunosuppressive function might be dependent on the FoxP3 expression level in Tregs. Chen et al. reported the detection of immune suppressive activities in HTLV-1 infected cell lines and primary ATLL cells [9]. Some reports also indicate that FoxP3 expression is associated with the immunosuppressive state of ATLL patients [14]. However, this relationship has not been observed in all cell lines, or in CD4^+^CD25^+^ cells from patients. Unlike in Tregs, patient-derived CD4^+^CD25^+^ cells did not exhibit immune suppressive function that paralleled FoxP3 expression levels. Furthermore, induced FoxP3 expression in HTLV-1-infected cell lines failed to trigger immunosuppressive activity. Notably, HTLV-1 infection also induces HTLV-1-associate myelopathy/tropical spastic paraparesis (HAM/TSP) and other autoimmune diseases. Some studies have reported decreased FoxP3 expression in the CD4^+^CD25^+^ cells of HAM/TSP patients compared with in healthy carriers [15]. However, the autoimmune disease severity is not proportional to the FoxP3 expression levels. Thus, it is now recognized that FoxP3 expression is not directly associated with immunosuppressive function.

Our present study started with analysis of the roles of molecules expressed in ATLL cells, which are associated with the immunosuppressive functions of Tregs. It is widely believed that cancer cells escape elimination by host immune systems by utilizing Tregs, or other immune suppressive systems, and many studies have been conducted to investigate the mechanism underlying this action. However, the precise immune functions of ATLL cells and the underlying mechanisms have not yet been elucidated. We detected CD39 expression on a portion of ATLL cells, with particularly high expression on aggressive subtypes. Thus, in the subsequent investigations in this study, we focused on CD39 and the related molecules CD73 and CD26.

CD39 (also termed ectonucleoside triphosphate diphosphohydrolase-1 or ENTPD1) is expressed or overexpressed in some types of neoplasms [16, 17], and is reportedly involved in the immunosuppressive mechanism via its extracellular adenosine triphosphate (ATP) metabolism. Thus, CD39 is now being investigated as a promising clinical target. CD39 is physiologically expressed on an effector/memory-like subset of FoxP3^+^ Tregs. It is an ectonucleotidase that catalyzes the hydrolysis of extracellular nucleotides, such as dephosphorylating ATP into AMP [18, 19]. CD73 (also termed ecto-5′-nucleotidase or NT5E) is a glycosyl phosphatidylinositol-linked membrane-bound glycoprotein that catalyzes the dephosphorylation of AMP into adenosine. CD73 is expressed on some subsets of B and T cells, dendritic cells, epithelial cells, and endothelial cells, including murine Tregs, but not human Tregs, and can exist in a soluble form in plasma. The ecto-enzymatic cascade of CD39 and CD73 generates extracellular adenosine that can prevent activation, proliferation, cytokine production, and cytotoxicity in T cells [18]. One of the suppressive mechanisms attributed to Tregs is metabolic disruption, which is partly ascribed to CD39 and CD73 expressions and the concomitant adenosine production [20–22]. Downstream of this enzymatic cascade, CD26 (also termed dipeptidyl peptidase VI or DPP4) interacts with adenosine deaminase (ADA) and inhibits the Tregs’ immunosuppressive function by catalyzing adenosine into inosine.

In this study, we investigated the expressions of CD39, CD73, and CD26 in leukemic cells (CD4^+^CD7^−^CADM1^+^ cells) and normal cells (CD4^+^CD7^+^CADM1^−^) from 40 patients, including 10 asymptomatic carriers of HTLV-1, and 30 ATLL patients. Through comparative genetic analyses, and in vivo and in vitro investigations using cell lines with altered CD39 expression, we clarified the mechanisms of immunosuppression by ATLL cells.

## Materials, patients, and methods

### Blood samples from patients

After obtaining informed consent, blood samples were collected from 10 asymptomatic HTLV-1 carriers and 30 ATLL patients (2 with smoldering type, 12 with chronic type, 14 with acute type, and 2 with lymphoma type). From these samples, we isolated peripheral blood mononuclear cells (PBMCs) using Ficoll-Paque (Pharmacia Biotech) density-gradient centrifugation.

### Antibodies, reagents, and cell lines

The HTLV-1-infected cell lines MT1, MT2, and MT4 were purchased from JCRB Cell Bank. The HTLV-1-infected cell line MJ, and the T-cell lymphoma cell lines Jurkat and EG7-OVA were provided by ATCC (VA, USA). All cell lines are identified based on short tandem repeat profiles by providers, and mycoplasma contaminations were denied both by providers and at our laboratories. Cell lines and PBMCs were cultivated using RPMI 1640 Medium (Nacalai Tesque, Kyoto, Japan) supplemented with 10% fetal bovine serum (FBS), and Penicillin–Streptomycin Mixed Solution (×100) (Nacalai Tesque). Mouse anti-CD4, CD8, CD7, CD39, CD73, and CD26 antibodies were purchased from Biolegend (San Diego, CA, USA), and anti-CADM1 antibody was purchased from MBL Inc. (Woburn, MA, USA). Adenosine 5′-triphosphate disodium salt hydrate, adenosine 5′-monophosphate hydrate, and adenosine were purchased from Sigma Aldrich (now Merck KGaA, Darmstadt, Germany). Polyinosinic-polycytidylic acd sodium salt (Poly(I:C)) was purchased from R&D systems (Minneapolis, MN, USA). The CellTrace^TM^ Violet Cell Proliferation Kit was purchased from Thermo Fisher Scientific (Waltham, MA, USA).

### FACS analysis and cell separation

PBMCs from ATLL patients were separated into CD4^+^CD7^−^CADM1^+^ ATLL cells and adjacent CD4^+^CD7^+^CADM1^−^ normal T cells using a fluorescence-activated cell sorter (FACS Aria; Becton, Dickinson and Company: BD, USA). These subsets were then subjected to total RNA sequencing experiments, and examinations of the expression patterns of CD39, CD73, and CD26 using flow cytometry (FACS Canto, BD).

### RNA sequencing and analysis

RNA sequencing analysis was performed as previously described [23]. Briefly, a library was constructed by using a SMARTer Ultra Low RNA kit (Clontech, Mountain View, CA, USA) to prepare amplified cDNA. Sequencing was performed on an Illumina HiSeq 2500 platform (Illumina, San Diego, CA, USA) in the 75-base single-end mode. Illumina Casava software (v1.8.2; Illumina) was used for base calling. The sequenced reads were mapped to human reference genome sequences (hg19) using TopHat (v2.0.13; https://ccb.jhu.edu/software/tophat/index.shtml) along with Bowtie2 (v2.2.3; http://bowtie-bio.sourceforge.net/bowtie2/index.shtml) and SAMtools (v0.1.19; http://samtools.sourceforge.net). We calculated the fragments per kilobase of exon per million mapped fragments using Cufflinks (v2.2.1; http://cole-trapnell-lab.github.io/cufflinks/). RNA-Seq data were analyzed based on the fold change between samples, calculated with a two-tailed Student’s *t* test (*P* < 0.1) using the Subio Platform and Subio Basic Plug-in (v1.20; Subio Inc., Aichi, Japan). Raw data were deposited in the Gene Expression Omnibus database (GSE127180).

### Measurements of ATP consumption, AMP, and adenosine synthesis

For measurement of ATP consumption, PBMCs isolated from subjects were resuspended at 2 × 10^6^ cells/mL, and added to a white 96-well plate (2 × 10^5^ cells/well). These cells were treated with 1 mM ATP for 1 or 3 h at room temperature. The remaining ATP concentration in the supernatant was measured using the ATPlite Luminescence System (PerkinElmer, Waltham, MA, USA). AMP and adenosine concentrations were measured using high-performance liquid chromatography (NANOSPACE; SHISEIDO Co. Ltd, Tokyo, Japan).

### Immunosuppression assays

Immunosuppression assays were performed as previously described [13]. Briefly, CellTrace^TM^ Violet (1 µM)-labeled responder CD4^+^ or CD8^+^ T cells (1 × 10^4^ cells) were cocultured with unlabeled tumor cells (1 × 10^4^), and were assessed for their proliferation. Cells were incubated in 96-well round-bottom plates, in RPMI medium supplemented with 10% FBS and Penicillin–Streptomycin Mixed Solution; and were stimulated with anti-CD3 (OKT3) and CD28 antibodies (0.5 μg/mL plate). After 72 h of culture, the proliferation of the CellTrace^TM^ Violet-labeled cells was assessed by flow cytometry.

### Generation of stable cell lines using CRISPR/CAS9 knockout systems and the expression vector

A CD39-specific CRISPR/CAS9 knockout system was purchased from Santa Cruz Biotechnology Inc. (Dallas, TX, USA). In accordance with the manufacturer’s instructions, Cas9 nuclease plasmid and three kinds of vectors for guide RNA sequences within CD39 were transfected into MJ cells using nucleofector. The disappearance of CD39 was confirmed by FACS analysis. For ectopic expression of CD39 into EG7-OVA, we used the pCMV3-C-GFPSpark® Vector (Sino Biological Inc., Beijing, China).

### Mice and in vivo tumorigenesis assay

For in vivo tumorigenesis assays, C57B/6 J mice were purchased from Clea Japan Inc. (Tokyo, Japan). Each EG7-OVA (CD39 expressing and control) cell sample (2 × 10^6^ cells) was suspended in PBS. Then the cell samples were subcutaneously injected into the flank on each side of six mice (8–10-week-old). Each male littermate was randomized in two groups. Tumor formation was monitored by palpation on postinjection days 6, 8, 10, and 13, and tumor volumes were calculated as length × width^2^/2. The tumors became palpable when their volumes reached 50 mm^3^ (4–5 mm in diameter). Injections were then performed in a new batch of mice, and immunostaining analysis was performed using the tumors from postinjection day 7. The staining procedure was conducted under the manufacturer’s protocol of Tyramide Signal Amplification Kit (Thermo Fisher Scientific). Briefly, each sample was incubated with a primary rabbit antimouse CD8 antibody, and the CD8 signal was amplified and was detected with Alexa Fluor^®^594 included in the kit. Cell nuclei were stained with 4′,6-diamidino-2-phenylindole (DAPI) (Nacalai Tesque).

All experimental procedures were conducted following protocols approved by the Institutional Animal Care and Use Committee of Osaka University.

### Statistical analysis

Data are presented as mean ± standard deviation (SD) from three or more independent experiments, and were compared using the paired two-tailed Student’s *t* test.

### Study approval

Studies involving human samples were approved by the Osaka University Hospital Institutional Review Board at Osaka University Hospital, Ryukyu University Hospital, and Kyushu Cancer Center, and met all requirements of the Declaration of Helsinki.

## Results

### Expressed RNAs associated with immunosuppressive function, compared between normal CD4^+^ cells and leukemic cells within ATLL patients

**Fig. 1:**
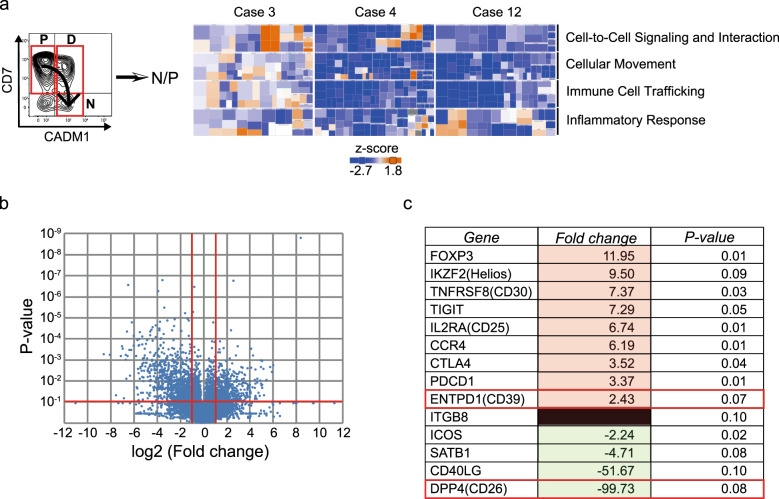
Transcriptome evaluation of CD4^+^ normal and leukemic cells in acute ATLL patients. **a** Left panel: CADM1 versus CD7 subpopulation plots for a patient with smoldering type ATLL. Section P of the plot contains CADM1^−^CD7^+^ cells (normal CD4^+^ cells); section D contains CADM1^+^CD7^+^ cells (indolent tumor cells); and section N contains CADM1^+^CD7^−^ cells (clonally expanded tumor cells). We separated the cells in areas P and N from one patient with acute and two with chronic ATLL, and compared the gene expressions. Right panel: Comparison of expressions of genes associated with inflammation and immunity. Color-coded heatmaps visualizing the differences in categorized gene expressions revealed that inflammation- and immunity-related genes were attenuated in ATLL cells. Blue and orange tiles, respectively, represent the genes with decreased and increased expression in progressing tumor cells, and the color strength indicates the degree. **b** Volcano plot showing comprehensive change in RNA expressions. Changes in individual gene expressions are compared between CD4^+^ normal cells and leukemic cells. Red lines divide genes according to increase or decrease (with 2- and 1/2-fold change) in expression level and *P* < 0.1 (Student’s *t* test). **c** List of the top ten upregulated and four downregulated genes associated with the immunosuppressive functions of Tregs ranked by *P* values, excluding CADM1 and CD7.

CD4^+^ cells in ATLL patients can reportedly be divided into four subgroups according to the expressions of CD7 and CADM1 [24]. Cells from peripheral blood from one patient with acute and two with chronic disease were sorted into normal cells and leukemic cells (Fig. 1a, left panel), and we used RNA-seq technologies to compare the whole transcriptome of leukemic cells with that of normal CD4^+^ cells. Leukemic cells showed attenuated expressions of immune- and inflammatory-associated genes (Fig. 1a, right panel). Figure. 1b shows a volcano plot indicating differentially expressed mRNAs between normal and leukemic cells. Among 23,284 mRNAs, 16,881 were detected in the three pairs of samples. A total of 1526 genes showed differential regulation by a fold change of >2.0 (*P* < 0.1), of which 547 were upregulated while 979 were downregulated in leukemic cells compared with normal cells (Supplementary Fig. S1). Figure 1c shows the top ten upregulated and four downregulated genes associated with Treg function, as ranked by *P* values, and excluding CADM1 and CD7. Among these genes, we focused on CD39 and CD26 because these molecules were recently identified as being involved in the regulation of extracellular adenosine, which has a strong anti-inflammatory function and plays a major role in Treg-mediated immunosuppression [25].

### Expressions of CD39, CD73, and CD26 in ATLL cells

**Fig. 2:**
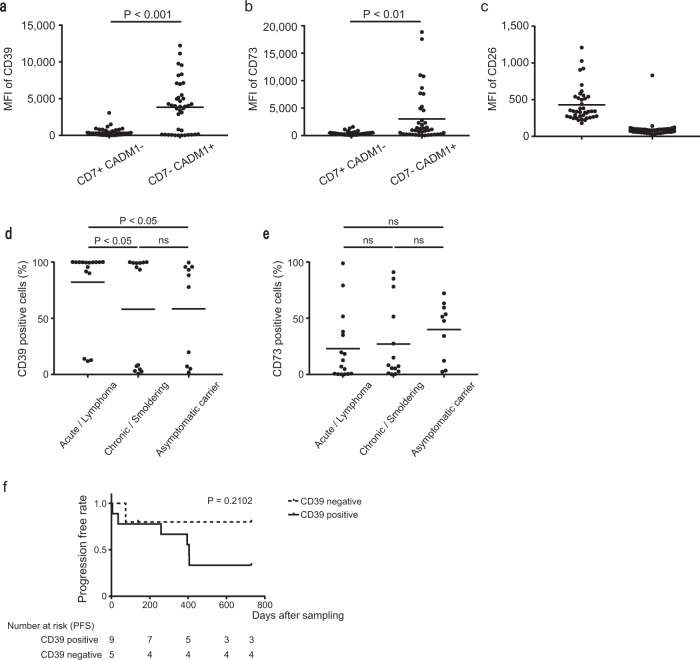
Expressions of CD39, CD73, and CD26 on various types of ATLL cells, and the correlation of CD39 expression with prognosis. **a**–**c** Plot of the mean fluorescence intensities (MFIs) of CD39, CD73, and CD26 on the cells in fraction P (CADM1^−^CD7^+^) and fraction N (CADM1^+^CD7^−^). Horizontal bars indicate median values. CD39 and CD26: *P* < 0.001, and CD73: *P* < 0.01. **d**, **e** Frequencies of CD39^+^ and CD73^+^ cells among CD4^+^ T cells within fraction N (CADM1^+^CD7^−^) from patients with acute/lymphoma, chronic/smordering subtypes of ATLL or HTLV-1 carrier. **f** Transformation-free survival of chronic/smoldering patients stratified by CD39 expression leukemic cells. Patients with chronic (*n* = 12) and smoldering (*n* = 2) ATLL were followed after blood collection up to a maximum of 2 years. Solid line indicates CD39^+^ patients, and dotted line indicates CD39^−^.

We investigated the expressions of CD39, CD73, and CD26 in leukemic cells (CD4^+^CD7^−^CADM1^+^ cells) and normal cells (CD4^+^CD7^+^CADM1^−^) from 40 patients, including 10 asymptomatic carriers of HTLV-1, and 30 ATLL patients (2 with smoldering type, 12 with chronic type, 14 with acute type, and 2 with lymphoma type) (Supplementary Table S1). These cases exhibited some variations in the expressions of CD39 and CD73 (Supplementary Fig. S2). We measured the mean fluorescence intensities of CD39, CD73, and CD26 in leukemic and normal cells. CD39 and CD73 expressions were significantly higher in leukemic cells than in normal cells (Fig. 2a, b). CD26 expression was not detected in leukemic cells (e.g., CD4^+^CD7^−^CADM1^+^ cells) from any patients, except one asymptomatic carrier, which was in accordance with effective Tregs in healthy donors. Moreover, all patients showed CD26 expression in normal cells (CD4^+^CD7^+^CADM1^−^ cells), in accordance with non-Treg helper T cells in healthy donors (Fig. 2c; Supplementary Fig. S3). We also compared the subsets of CD39^+^ and CD73^+^ leukemic cells between patients with aggressive and indolent ATLL subtypes. In patients with aggressive ATLL, most leukemic cells were CD39^+^, while the ratios of CD39 expressing leukemic cells in chronic/smoldering patients or in asymptomatic carriers are significantly lower than in patients with aggressive type (Fig. 2d). On the other hand, the ectopic expression of CD73 did not significantly differ among these subtypes (Fig. 2e). We also observed the patients with chronic disease prospectively after the sampling time. Within the 2-year observation period, acute transformations were experienced by 67% of CD39^+^ patients and 20% of CD39^−^ patients (Fig. 2f). Although we could not obtain significant difference between two groups within the limited period and in the limited number of patients, a trend that the patients with CD39^+^ disease have worse prognosis could be observed. These results suggested that CD39 expression was associated with disease severity.

### Extracellular ATP metabolism by CD39^+^ leukemic cells

**Fig. 3:**
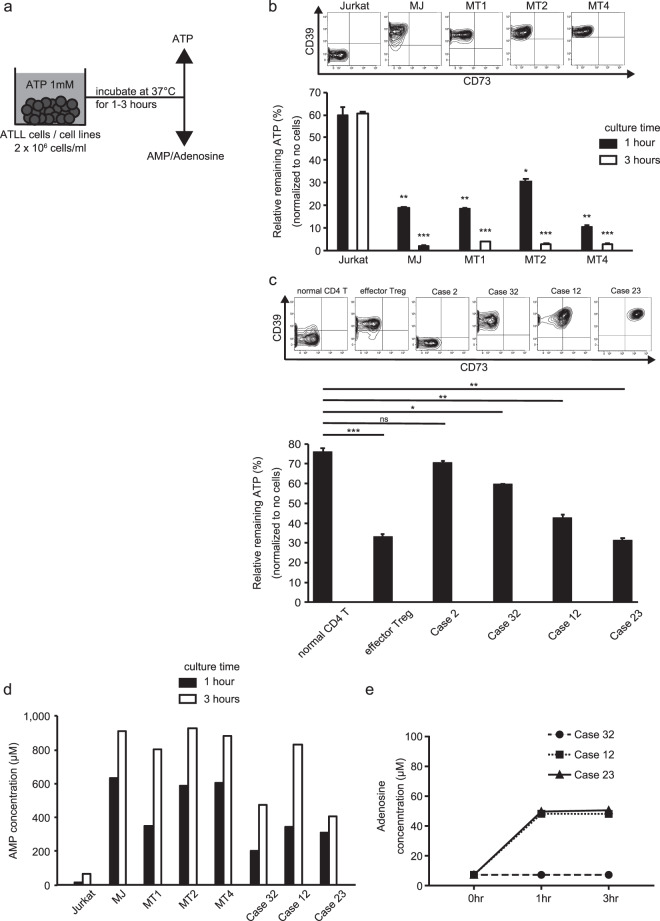
Extracellular ATP metabolism through CD39 and CD73 expressed on HTLV-1 infected cell lines and patients’ leukemic cells. **a** The experimental scheme. Cell lines and patients’ leukemic cells were cultured for 1 or 3 h in 10% FCS RPMI supplemented with 1 mM ATP, and then supernatants were subjected to ATP or AMP/adenosine measurement. **b** Contour plots in the upper panel show CD39 and CD73 expression on T-cell lines. Flow cytometry was used to examine the expressions of CD39 and CD73 on the Jurkat cell line, human T-cell lymphoma (other than ATLL) cell line, and the MJ, M1, M2, and M4 HTLV-1 infected cell lines. Bar graph in the lower panel shows the remaining extracellular ATP concentration after culture for the indicated hours. Bar heights indicate the mean ± SD of the % ratio of remaining ATP to the supplemented amount from triplicate experiments. *P < 0.05; **P < 0.01; ***P < 0.001, Student’s *t* test, compared with the value of Jurkat. **c** The same experiments as in b using primary CD4^+^ T cells. Lower panel shows relative remaining ATP concentrations after 1 h of incubation. Data significance was evaluated compared with the value of the normal CD4^+^ T cells. **d** AMP concentrations after 1 h (black) or 3 h (white) of incubation are shown. **e** Adenosine concentrations after the culture of CD39-expressing and/or CD73-expressing ATLL leukemic cells. Leukemic cells from Case 32 express CD39 but not CD73, and those from Cases 12 and 23 express both CD39 and CD73.

Next, we examined the roles of CD39 and/or CD73 in ATLL cells by observing the reduction rates of ATP loaded in culture medium, and the synthesis of AMP/adenosine. As previously reported, extracellular ATP is converted into AMP by CD39, and AMP is subsequently catalyzed into adenosine by CD73. ATLL leukemic cells from patients or from HTLV-1-infected cell lines were incubated in culture medium supplemented with 1 mM ATP, at 37 °C under 5% CO_2_ (Fig. 3a). After 1 or 3 h of incubation, one removed aliquot was subjected to ATP consumption measurement, and another to AMP/adenosine synthesis measurement. Extracellular ATP was rapidly reduced and AMP accumulated in the presence of effector Tregs from healthy donors, or HTLV-1 infected cell lines (MJ, MT1, MT2, and MT4) or leukemic cells from patients expressing CD39 (Cases 32, 12, and 23). In contrast, in the presence of non-Treg-Th cells, the T-cell line (Jurkat), or CD39^−^ leukemic cells from patients (Case 2), about 60% of the supplemented quantity of ATP remained (Fig. 3b, c). Furthermore, adenosine was rapidly synthesized in the presence of CD73-expressing cells (Cases 12 and 23) (Fig. 3d), while adenosine concentrations were below the detection rate (7.2 μM/L) in the presence of CD73^−^ cells (Fig. 3e).

### Effects of CD39 inhibitors and CD39 knockout in an HTLV-1-infected cell line on extracellular ATP metabolism and its immunosuppressive activity

**Fig. 4:**
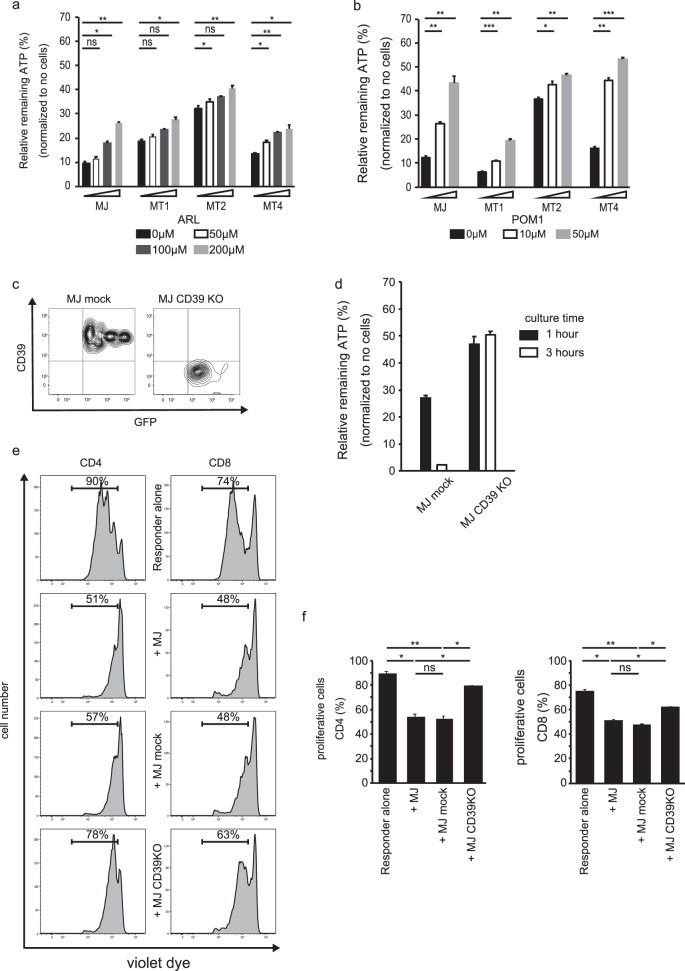
Effects of CD39 blocking or knockout on ATP metabolism and on the immunosuppressive effects of HTLV-1-infected cell lines. **a**, **b** Each cell line was cultured with 1 mM ATP, with or without the CD39 inhibitors ARL (left panel) or POM1 (right panel), for 1 h at the indicated concentrations. All cell lines were CD39 positive. Results are expressed as the mean ± SD of at least three independent experiments. **c** The CD39-positive cell line MJ was introduced with empty vector (mock) or CRISPR/CAS9 knockout plasmid including a GFP expression site. Flow cytometry confirmed CD39 knockout in the GFP-positive fraction of CD39KO transfected cells, and CD39 expression in mock transfected cells. The GFP-positive cells were selected using a FACS cell sorter and were used in the following experiments. **d** As in Fig. 3, ATP consumption was measured in mock or CD39KO transfected MJ cells. Bar heights indicate the mean ± SD of the % ratio of remaining ATP relative to supplemented amount, from triplicate experiments. **e** Suppressive activity of mock/CD39KO MJ cells. CellTrace^TM^ Violet-labeled CD25^−^CD4^+^ (left panels) or CD8^+^ T cells (1 × 10^4^) were stimulated with soluble anti-CD3 and anti-CD28 mAbs in the presence of irradiated PBMCs excluding CD3 positive cells. Mock or CD39KO MJ cells (1 × 10^4^) were added to the cultures. After 3 days of culture, the violet intensity was measured by FACS as an indicator of cell division. **f** Bar charts show the frequency (%) of responder T cells, which have undergone cell division, with or without MJ cells or with each transfected MJ cell line. The results are expressed as the mean ± SD of triplicate experiments. For **a**, **b**, and **f**, data were compared using Student’s *t* test; ns not statistically significant; *P < 0.05; **P < 0.01; and ***P < 0.001.

The two CD39 inhibitors ARL and POM1 both dose dependently altered the ATP reduction by HTLV-1-infected cell lines (Fig. 4a, b). We also generated a CD39 knockout cell line from MJ cells, using the CRISPR/Cas9 plasmid systems. CD39 expression was completely eliminated in all GFP^+^ cells induced with CRISPR/Cas9/CD39, but not in mock MJ cells (Fig. 4c). We isolated GFP^+^CD39 knockout and GFP^+^ mock MJ cells, and subjected them to ATP consumption measurement. As in the previous experiments, each cell line was supplemented with 1 mM ATP and, after 1 or 3 h of incubation, the ATP concentrations in supernatants were measured. In the presence of mock MJ cells, extracellular ATP was rapidly reduced and almost exhausted in 3 h. In contrast, in the presence of CD39 knockout cells, extracellular ATP was maintained even after 3 h (Fig. 4d).

To assess the immunosuppressive activities of these cell lines, CD39 knockout or mock cells were cocultured with human CD4^+^ or CD8^+^ responder cells from healthy donors that were fluorescently labeled with violet dye. After 3 days of culture, we traced the proliferation of CD4^+^ or CD8^+^ normal T cells by dilution of the fluorescent dye. The proliferation of CD4^+^ and CD8^+^ T cells was significantly inhibited by coincubation with MJ and mock-induced MJ cells, but not by CD39 knockout MJ cells (Fig. 4e, f). In other words, CD39 knockout almost entirely negated the immunosuppressive activity of the HTLV-1-infected cell line.

### Effects of induced CD39 expression in a CD4^+^ T-cell line on the immune response against tumorigenesis in vivo

**Fig. 5:**
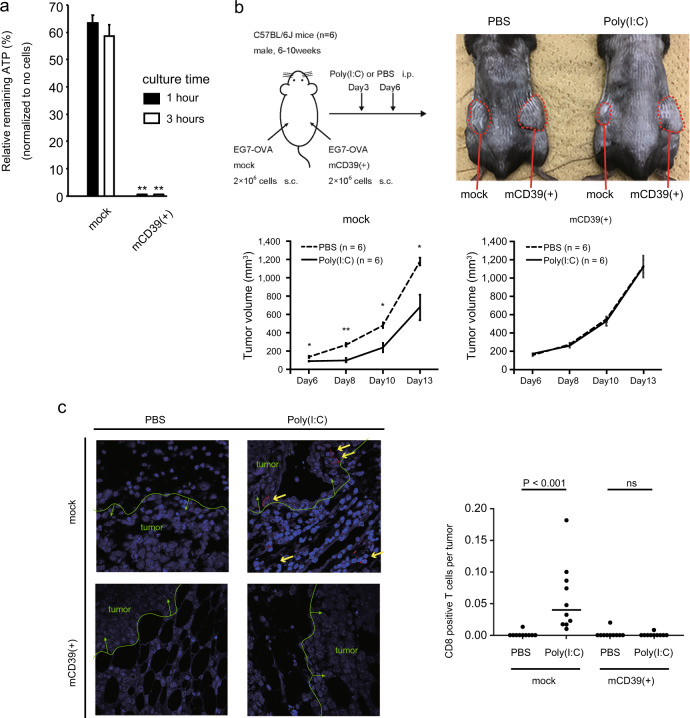
Effects of forced CD39 expression in a CD39^−^ T-cell line on ATP metabolism and antitumor immunity in vivo. The CD39^**−**^ murine T lymphoblast cell line EG7-OVA was infected with a retrovirus vector expressing murine CD39 or mock, including a GFP-expressing site. **a** As in Fig. 3, after culture for the indicated number of hours, ATP consumption was measured in mock or CD39 transfected EG7-OVA cells. Bar heights indicate the mean ± SD of the % ratio of remaining ATP relative to the supplemented amount, from triplicate experiments. **P < 0.01, Student’s *t* test, compared with the value of mock transfected EG7-OVA. **b** Upper left panel: The experimental scheme of the tumorigenesis assay. We subcutaneously transplanted mock infected EG7-OVA cells (2 × 10^6^) into the left flanks of C57B/6 J mice, and the same amount of CD39-transfected EG7-OVA cells into the right flanks. On posttransplantation days 3 and 6 the mice were intraperitoneally injected with Poly(I:C) or PBS. Tumor sizes were observed from posttransplantation day 6.Upper right panel: Photographic image of typical tumors on Day 13. Tumors in the left flanks are from transplanted CD39^−^ EG7-OVA cells and those in the right flanks from transplanted CD39^+^ cells. PBS was administered to the left mouse and Poly(I:C) was administered to the right. Lower panels: Line chart indicates volumes of mock infected tumors (left panel; left flanks) and CD39^+^ tumors (right panel; right flanks). Dotted and continuous lines, respectively, indicate the tumor volumes in PBS-injected mice and Poly(I:C)-injected. The results are expressed as the mean ± SD from six mice. *P < 0.05 and **P < 0.01, Student’s *t* test comparing the values in PBS-injected and Poly(I:C)-injected mice. **c** Left panels: Immunostaining of each tumor sample. CD8 positive cells were stained with Alexa Fluor®594 and cell nuclei with DAPI. Yellow arrows indicate CD8^+^ cells. Green lines show the tumor borders. Right panels: CD8^+^ cells observed in each tumor sample. Plot shows the proportion of CD8^+^ cells to DAPI-stained cells in the boundary areas of tumors in each group of mice. Cell numbers were counted in each field of view. Horizontal bars indicate median values. *P* < 0.001.

To further assess the function of CD39 in the immunosuppressive activity of ATLL cells, we also stably induced murine CD39 into the EG7-OVA T-cell lymphoma cell line, which originally did not express CD39. In mice, EG7-OVA cells induce major histocompatibility complex class I restricted responses to cytotoxic T lymphocytes, which can be effectively stimulated by Poly(I:C) through Toll-like receptor. CD39 expression in GFP^+^ cells was confirmed by flow cytometry (Supplementary Fig. S4). ATP consumption experiments revealed that CD39-induced cells almost entirely exhausted the supplemented ATP within 1 h, while mock-induced cells left over 60% of the supplemented ATP even after 3 h, as was previously observed in Jurkat cells (Fig. 5a).

We subcutaneously transplanted the CD39^+/−^ EG7-OVA cells into murine flanks: CD39^−^ EG7-OVA cells into the left flank, and CD39^+^ cells into the right flank. On posttransplantation days 2 and 5, we intraperitoneally administered either Poly(I:C) or PBS. From posttransplantation days 5 to 12, the tumor sizes were observed (Fig. 5b upper left panel). Figure. 5b upper right panel shows photographic images of the tumors; the tumor of mock cells in the left flank of a mouse administered with Poly(I:C) (image on the right) appeared smaller than that in the mouse administered with PBS (image on the left), while the tumors of CD39^+^ EG-OVA cells in the right flanks were the same size in both mice. Tumor immunity induced by Poly(I:C) effectively reduced the sizes of mock transplanted tumors, while the sizes of CD39^+^ tumors were completely unaffected by Poly(I:C) administration (Fig. 5b lower panels). These results indicated that CD39 expression completely negated the tumor immunity induced by Poly(I:C).

To elucidate the state of tumor immunity during this process, we further performed immunostaining assays using a specimen of each tumor from the mice. CD8^+^ cytotoxic T cells were observed to invade around the CD39^−^ tumors upon Poly(I:C) administration, which was not observed in the CD39^+^ tumors (Fig. 5c).

Overall, our findings demonstrated that ATLL cells expressed CD39 at a high rate, and ectopically expressed CD73 in some cases. Our results further suggested that this enabled ATLL cells to escape the antitumor immunity of environmental cells via inhibition of the host immune response through the extracellular ATPDase-Adenosine cascade, including CD39 and their own or surrounding CD73.

## Discussion

In many kinds of neoplasms, especially solid tumors, large numbers of FoxP3^+^ Tregs infiltrate into tumors [26, 27]. It is likely that Tregs, which are naturally engaged in self-tolerance, concurrently hinder anticancer immune surveillance in healthy individuals and inhibit the development of effective antitumor immunity in tumor-bearing patients [28]. Increased numbers of Tregs, and decreased ratios of CD8^+^ T cells to FoxP3^+^ Tregs, infiltrating into a tumor are reported to be strongly correlated with poor prognosis in various cancers [26, 28, 29]. Thus, research has been focused on the derivation of antitumor immunity and control of Tregs for effective cancer treatment. In our present study, we found that in addition to effector Tregs, CD39^+^ ATLL cells themselves have immunosuppressive function, which appears to allow leukemic cells to overcome and escape from host antitumor immunity, thus promoting the disease.

ATLL patients are known to be susceptible to various opportunistic infections, including pneumocystis pneumonia, fungal infections, and herpes virus disease, due to defective cellular immunity [9, 30, 31]. The causative mechanism of this immunosuppressive state in ATLL patients is complicated. Although the immune suppression is largely due to the patients’ decrease of normal T cells, ATLL patients exhibit a much more severe immunocompromised state than patients with other T-cell leukemias. Notably, the majority of ATLL leukemic cells have a phenotype the same as or similar to that of normal Tregs. This has prompted discussion of whether ATLL leukemic cells themselves have immunosuppressive function, which has remained controversial. In ATLL, leukemogenesis is initiated by HTLV-1 infection, but those infected cells are transformed into ATLL leukemic cells through at least five steps of genomic and/or epigenetic changes [32, 33]. Thus, ATLL leukemic cells exhibit a relatively wide variety of characteristics between different patients. It is now widely believed that leukemic cells have immunosuppressive potential in at least some cases of ATLL, but not necessarily all cases.

We clarified that the majority of aggressive ATLL cells express CD39, which promotes adenosine accumulation and provides immunosuppressive function to ATLL cells. These data, together with other in vitro results, indicate that the CD39-mediated immune control in CD39^+^ ATLL cells will greatly contribute to the progression of ATLL disease. In our present study, we intended to examine the immunosuppressive function and the effects on the growth of tumor of CD39 expressed on ATLL leukemic cells. In order to exclude the effects of other factors, we ectopically expressed or knocked out CD39 in ATLL or other cell lines, and exaggerated the effects of loss and gain of CD39. Although biological immunosuppressive activities of primary ATLL cells derived from patients were not directly examined, we confirmed that CD39 expressed on ATLL cells from patients have full function as an extracellular ATPDase exactly in the same way as in these CD39-expressing cell lines. The final player of immunosuppression is adenosine, which is produced under the CD39’s ATPDase cascade. Therefore, it can be educed that CD39-expressing ATLL primary cells themselves have immunosuppressive function just as CD39-expressing cell lines.

Among the cases with CD39^+^ ATLL cells, CD73^+^ cells were relatively rare, and in vitro experiments revealed that CD39^+^ cells lacking CD73 expression did not produce adenosine. However, in vivo, ATLL cells will be surrounded by abundant CD73-expressing cells, including B cells, T cells, and dendritic cells [34]. In addition, CD73^+^ exosomes will rapidly dephosphorylate the AMP catalyzed by CD39 into adenosine [35]. Moreover, CD26 expression was apparently downregulated in leukemic cells compared with in normal CD4 cells. Since CD26 acts as an adenosine decomposer in cooperation with ADA, the reduction of CD26 would result in adenosine accumulation. Thus, the adenosine accumulation around leukemic cells that is induced by the high CD39 expression is further enhanced by the lack of CD26, which actively suppresses the cell immunity surrounding ATLL cells, producing a favorable environment for their survival. Recently, some papers reported that in chronic lymphocytic leukemia (CLL) patients, CD39-expressing nonmalignant T lymphocytes are increased compared with in normal control, and the higher expression levels of CD39 are associated with the worse prognosis of the disease [16, 36]. This information would support our results that CD39 expression on ATLL cells themselves correlates with the progression of disease through the suppression of tumor immune in the host. On the other hand, the expression of CD73 on nonmalignant T cells was reduced and its expression correlates with the better prognosis in CLL. Authors explain that the expression of CD73 would raise the responsibility to chemotherapy, such as Fludarabine. In our study, CD73 was detected on ATLL cells only in a few patients, and could not detect the correlation with the prognosis. However, in our data, CD73 was essential for the adenosine synthesis under CD39, and we expect that CD73 also play an important role in the immunosuppressive function of ATLL cells.

To determine whether the expression of CD39 could be some clue to predict the prognosis of the patients, some extensive and long-term study must be performed. On the other hand, mechanisms for the inhibition of antitumor immunity by CD39/CD73 have been reported in various neoplasms [37, 38], and several clinical trials targeting CD39/CD73 have already been conducted. ATLL is poorly responsive to chemotherapies and has an extremely dismal prognosis. The anti-CCR4 antibody mogamulizumab was recently developed [39, 40] and several therapeutic strategies are currently being tested, including immunostimulants, such as interferon and anti-PD-1/PDL-1 antibodies. Targeting CD39, as the main factor in antitumor immunity inhibition in ATLL cells, will be a promising clinical strategy for ATLL treatment.

## Supplementary information

FigureS1

FigureS2

FigureS3

FigureS4

Tbale S1
