# Supplementary material for: Ectonucleotidase CD39 is highly expressed on ATLL cells and is responsible for their immunosuppressive function
Source: Leukemia. 2020 Mar 20;35(1):107–18. doi: 10.1038/s41375-020-0788-y (PMC7787980; doi:10.1038/s41375-020-0788-y)
Supplement: Supplementary file 1 — FigureS1 [file 41375_2020_788_MOESM1_ESM.pptx]

## Slide 1
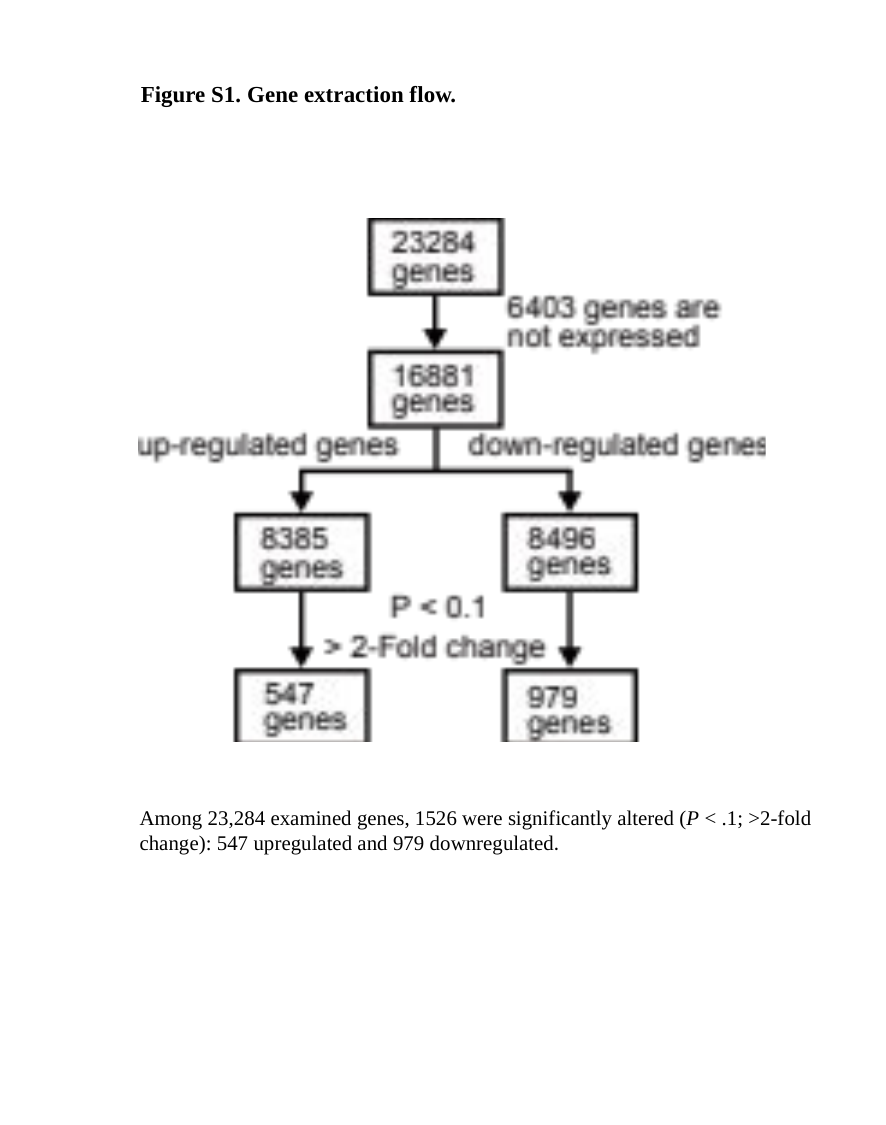

Figure S1. Gene extraction flow.
Among 23,284 examined genes, 1526 were significantly altered (P < .1; >2-fold change): 547 upregulated and 979 downregulated.
