# Supplementary material for: Ectonucleotidase CD39 is highly expressed on ATLL cells and is responsible for their immunosuppressive function
Source: Leukemia. 2020 Mar 20;35(1):107–18. doi: 10.1038/s41375-020-0788-y (PMC7787980; doi:10.1038/s41375-020-0788-y)
Supplement: Supplementary file 2 — FigureS2 [file 41375_2020_788_MOESM2_ESM.pptx]

## Slide 1
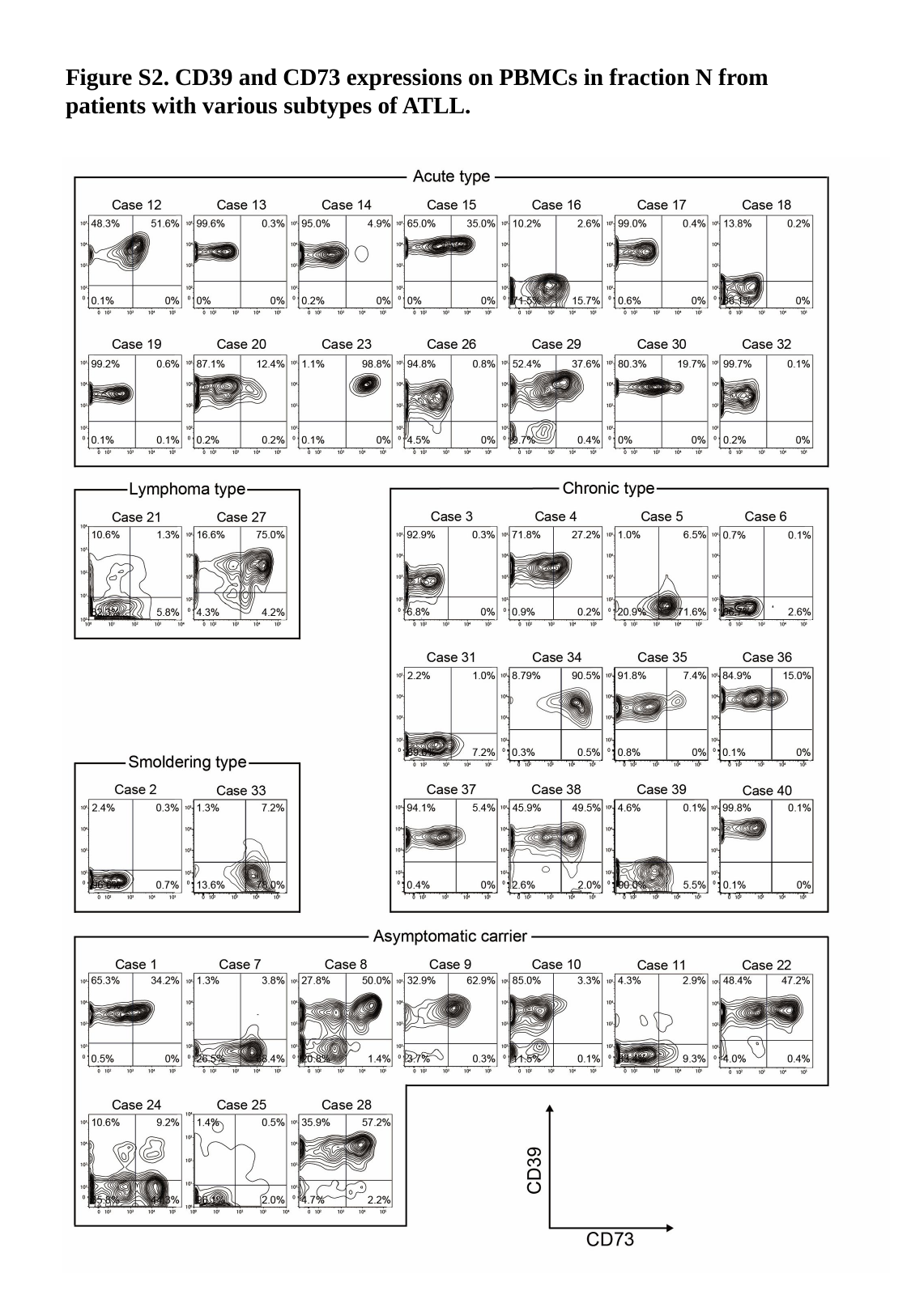

Figure S2. CD39 and CD73 expressions on PBMCs in fraction N from patients with various subtypes of ATLL.
