# Supplementary material for: Ectonucleotidase CD39 is highly expressed on ATLL cells and is responsible for their immunosuppressive function
Source: Leukemia. 2020 Mar 20;35(1):107–18. doi: 10.1038/s41375-020-0788-y (PMC7787980; doi:10.1038/s41375-020-0788-y)
Supplement: Supplementary file 3 — FigureS3 [file 41375_2020_788_MOESM3_ESM.pptx]

## Slide 1
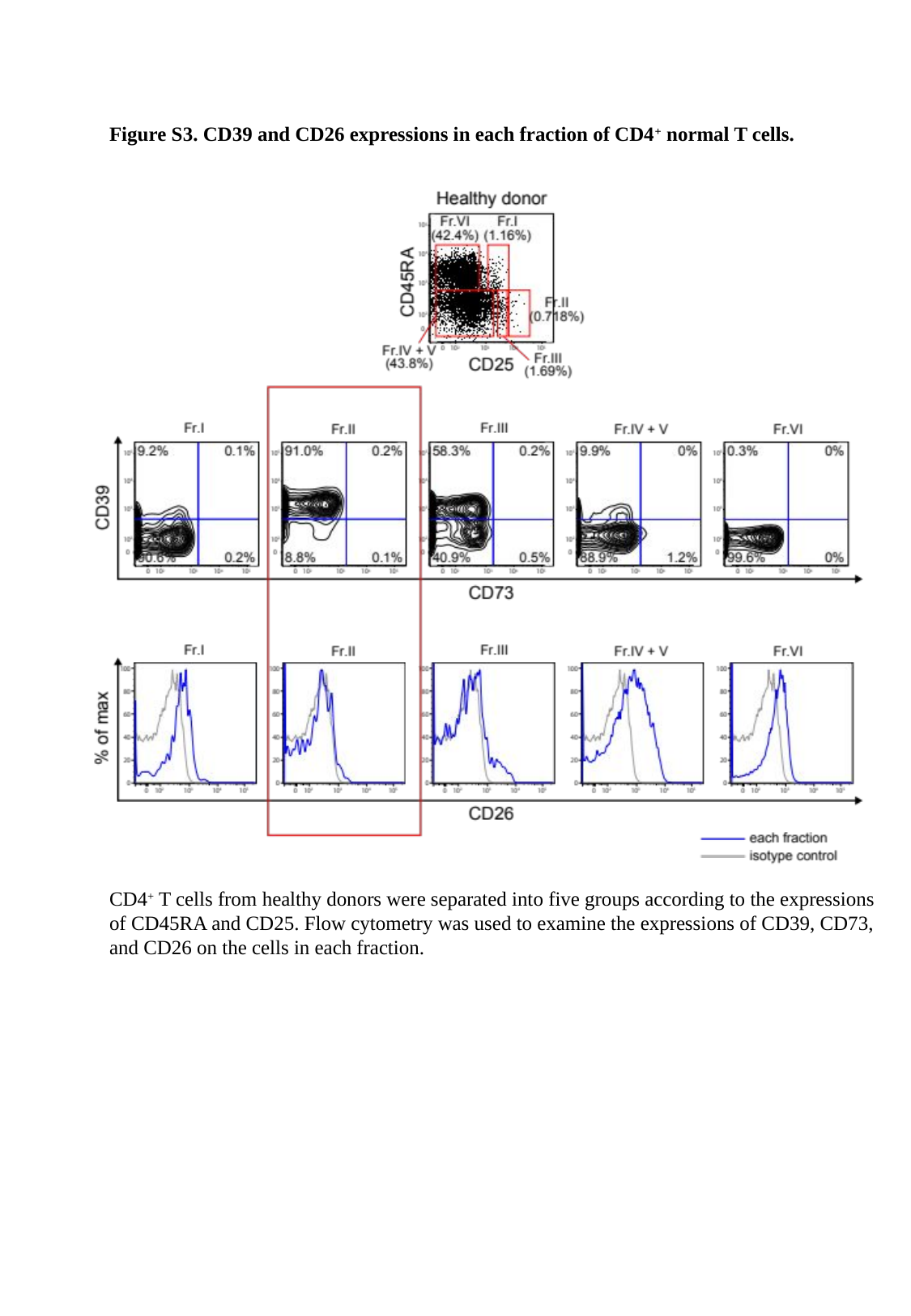

Figure S3. CD39 and CD26 expressions in each fraction of CD4+ normal T cells.
CD4+ T cells from healthy donors were separated into five groups according to the expressions of CD45RA and CD25. Flow cytometry was used to examine the expressions of CD39, CD73, and CD26 on the cells in each fraction.
