# Supplementary material for: Ectonucleotidase CD39 is highly expressed on ATLL cells and is responsible for their immunosuppressive function
Source: Leukemia. 2020 Mar 20;35(1):107–18. doi: 10.1038/s41375-020-0788-y (PMC7787980; doi:10.1038/s41375-020-0788-y)
Supplement: Supplementary file 4 — FigureS4 [file 41375_2020_788_MOESM4_ESM.pptx]

## Slide 1
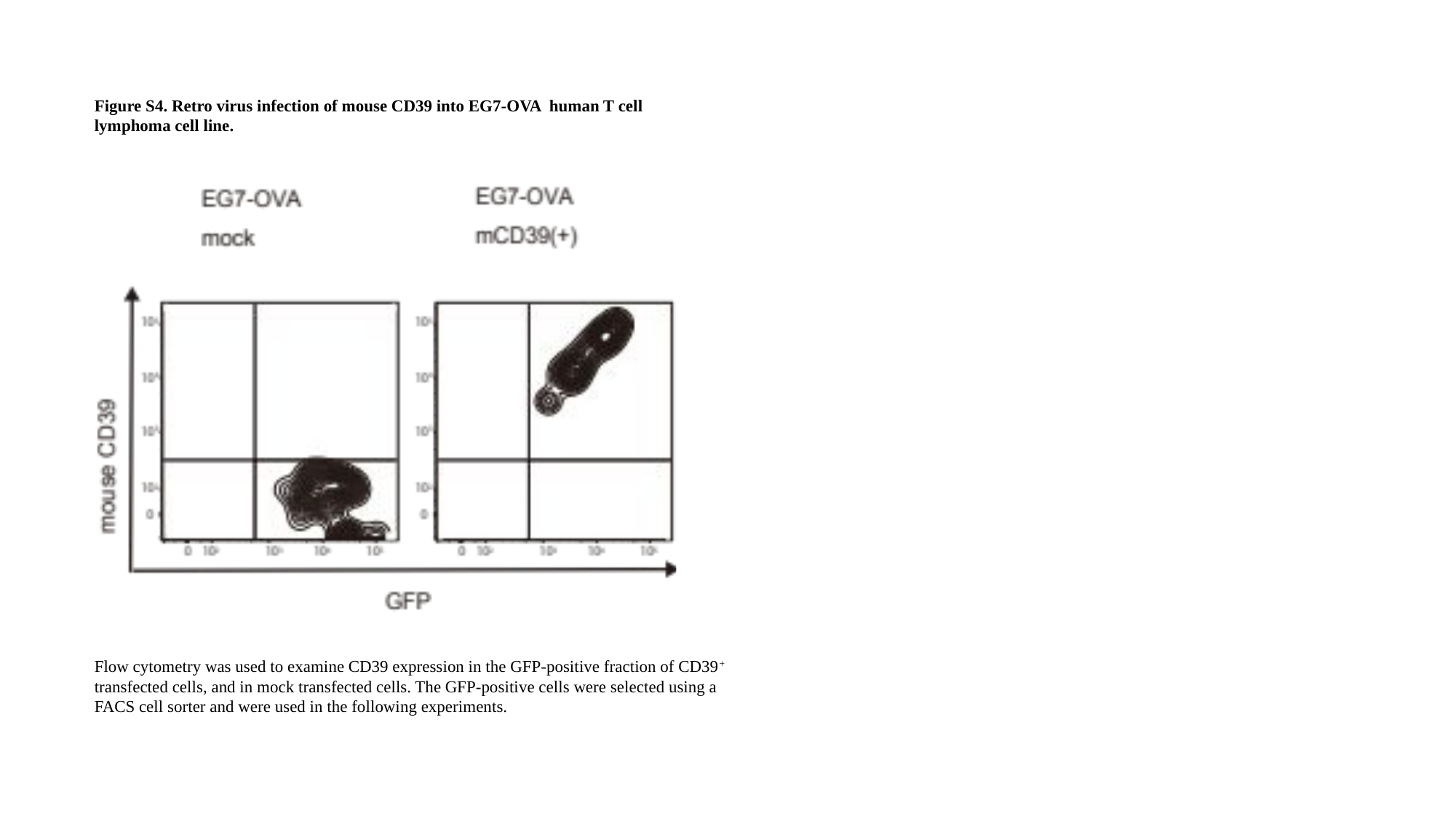

Figure S4. Retro virus infection of mouse CD39 into EG7-OVA human T cell lymphoma cell line.
Flow cytometry was used to examine CD39 expression in the GFP-positive fraction of CD39+ transfected cells, and in mock transfected cells. The GFP-positive cells were selected using a FACS cell sorter and were used in the following experiments.
