# Supplementary material for: Ectonucleotidase CD39 is highly expressed on ATLL cells and is responsible for their immunosuppressive function
Source: Leukemia. 2020 Mar 20;35(1):107–18. doi: 10.1038/s41375-020-0788-y (PMC7787980; doi:10.1038/s41375-020-0788-y)
Supplement: Supplementary file 5 — Tbale S1 [file 41375_2020_788_MOESM5_ESM.pdf]

Table S1. Clinical background of patients.

	Total (n=40)
Males / Females	15/25
Age (year), median (range)	66 (34-85)
Clinical type	
Acute	14 (35.0%)
Lymphoma	2 (5.0%)
Chronic	12 (30.0%)
Smoldering	2 (5.0%)
Asymptomatic carrier	10 (25.0%)

This study enrolled a total of 30 patients with various subtypes of ATLL.
